# Study on Maternal SNPs of MTHFR Gene and HCY Level Related to Congenital Heart Diseases

**DOI:** 10.1007/s00246-020-02449-1

**Published:** 2020-11-21

**Authors:** Hui Shi, Shiwei Yang, Ning Lin, Peng Huang, Rongbin Yu, Mei Chen, Lijuan Wang, Zhixin Jiang, Xiaoru Sun

**Affiliations:** 1 Jiangsu Institute of Planned Parenthood Research, Nanjing, 210036 Jiangsu China; 2 Department of Cardiology, Children’s Hospital of Nanjing Medical University, Nanjing, 210008 China; 3 Nanjing Medical University, Nanjing, 210029 Jiangsu China

**Keywords:** Congenital heart disease, SNPs of MTHFR, Homocysteine

## Abstract

The aim of this study is to evaluate the relationship between maternal single nucleotide polymorphisms (SNPs) of methylenetetrahydrofolate reductase (MTHFR) gene with plasma homocysteine (HCY) level and offspring congenital heart diseases (CHDs). 338 mothers with offspring CHDs as case group and 306 mothers of normal children as control group were recruited. Their pregnant histories were interviewed by questionnaire and the MTHFR rsl801133 and rsl801131 were genotyped. The case–control analysis was used to find out the relationship between maternal SNPs of MTHFR gene and offspring CHDs. And the plasma HCY concentration of the mothers of CHDs children was detected. This case–case study was intended to find out the relevance between maternal HCY level and SNPs of MTHFR gene. There were significant differences in the gender of children, occupation of mothers, family history with CHDs, history of abortion, history of adverse pregnancy, early pregnancy health, fetus during pregnancy, pesticide exposure and drug exposure in CHDs group and control group (*P* < 0.05). MTHFR rs1801133 was significantly associated with their offspring CHDs in mothers. The polymorphism of maternal MTHFR rs1801133 increased plasma HCY level, especially the homozygous mutation. Besides the environmental factors, our results suggested that the maternal MTHFR rs1801133 polymorphism might be a risk factor of their offspring CHDs, which may be due to the hyperhomocysteinemia by abnormal metabolism of HCY.

**Electronic supplementary material:**

The online version of this article (10.1007/s00246-020-02449-1) contains supplementary material, which is available to authorized users.

## Introduction

Congenital heart diseases (CHDs) refers to abnormal cardiovascular structure or function at birth. Monitoring data on birth defects in China showed that CHDs have been the highest incidence of birth defects and increased yearly since 2005 [1]. They became the first cause of death among 1 year infants in China due to severe illness, multiple complications and high mortality [2]. The etiology of CHDs is not fully understood. A great amount of studies indicated CHDs are affected by both genetic and environmental factors [3, 4]. Methylenetetrahydrofolate reductase (MTHFR), the major enzymes in the folate/homocysteine (HCY) metabolism pathway, catalyzes the conversion of 5,10-methylenetetrahydrofolate into 5-methyltetrahydrofolate. The mutation of the MTHFR gene results in the decrease of the enzyme activity and the synthesis of methionine, the direct precursor of *S*-adenosylmethionine (SAM) which is an important methyl donor in cells participating in the tissue development and growth. Most common genetic variants of the MTHFR gene are the 677C > T polymorphism (rs1801133) and 1298A > C polymorphism (rs1801131), which result in the conversion of alanine to valine and glutamate to alanine, respectively. In our previous study, we found environment risks for CHDs in Chinese and the maternal genotype in MTHFR rs1801131 variant significantly increased offspring CHDs risk by screened five single nucleotide polymorphisms (SNPs) of three genes [5]. However, the relationship between the maternal plasma HCY level and SNPs of MTHFR was only showed a tendency without statistic significances. The objective of the present further study was to evaluate the relationship between the genotype and HCY level in the plasma of mothers with offspring CHDs by case–case study in a larger crowd, and meanwhile a further investigation on maternal environmental risks and the genotype factor in MTHFR by case–control study.

## Materials and Methods

### Ethics Statement

The study had been approved by the Ethics Review Committee of the Jiangsu Institute of Planned Parenthood Research, Nanjing, China. Prior written informed consent was obtained from all the participants enrolled in the study.

### Participants

From May 2012 to September 2016, the mothers of CHDs children in the study were recruited from the Nanjing Children’s Hospital, and the mothers of healthy children with matching age were recruited from the preconception health care center, Jiangsu Province, China. They were interviewed face-to-face to collect personal information and excluded diseases such as CHDs, hypertension, diabetes, tumor and others. Each participant donated 3 mL venous blood for HCY tests and host DNA genotyping.

### DNA Collection and Genotyping

Genomic DNA was extracted from a leukocyte pellet by traditional proteinase K digestion and followed by phenol–chloroform extraction and ethanol precipitation. MTHFR rs1801133 and rs1801131 were genotyped by the Taq-Man allelic discrimination assay on an ABI 7900 system (Applied Biosystems, La Jolla, CA). The information on primers and probes are shown in Table S.

### Detection of HCY

Plasma was separated from venous blood with EDTA anticoagulant in time. HCY tests were performed by circulatory enzyme method on Backman automatic biochemical analyzer in Clinical Laboratory of Zhongda hospital southeast university. The HCY detection were performed according to the manufacturer’s (NingBo Medical System Biotechnology Co., Ltd) protocols briefly as follows. The sample was mixed with reagent 1 and incubated at 37 °C for 5 min. Then the mixture was added reagent 2, and incubated at 37 °C for another 2 min. The absorbance change of the sample was monitored for 3 min under the detection wavelength of 340 nm (main)/405 nm (auxiliary), and the HCY concentration was calculated by the formula.

### Statistical Analysis

The survey data were recorded independently by two individuals and were checked after entry. Counting variables were described by frequency (percentage), and their differences between groups were tested by *χ*^2^ test. Quantitative data were described by means ± standard deviation (mean ± SD), their difference between groups was tested by *t*-test or One-Way ANOVA. We used median (25% and 75% interquartile) to describe skewness distribution variables, and their difference between groups was tested by rank sum test. Odds ratios (ORs) and their 95% confidential intervals (CIs) were calculated as a measure of difference in the response rate using logistic regression analysis. Multivariate logistic regression was used to analysis the association of selected SNPs with offspring CHDs, in which factors showing significant differences in univariate analysis were included for adjustment.

The statistical analyses were performed using Statistical Analysis System software (version 9.1.3, SAS Institute, Cary, NC). All *P* < 0.05 in a two-sided test was considered statistically significant.

## Results

In this study, a total of 338 mothers with offspring CHDs and 306 mothers of normal children with available blood sample were included. The selected characteristics of the mothers in CHDs group and the controls in the study are shown in Table 1. There were significant differences in the gender of children, child-bearing age, education, occupation, history of abortion, early pregnancy health, smoking, pesticide exposure and drug exposure in two groups (*P* < 0.05 for both comparisons).

**Table 1:** Demographic and selected variables in mothers with offspring CHDs and controls

Variables	Control *N*(%)	Case *N*(%)	*P*
Gender of children^a^			< 0.001
Male	171(57.00)	138(40.95)	
Female	129(43.00	199(59.05)	
Child-bearing age^a,b^			
24.12 ± 5.11	25.93 ± 2.24	26.52 ± 5.25	0.075
< 25	90(31.25)	150(45.18)	< 0.001
≥ 25	198(68.75)	182(54.82)	
Education^a^			< 0.001
Middle school and lower	110(39.71)	192(56.97)	
High school and above	167(60.29)	145(43.03)	
Occupation^a^			0.005
Farmer	44(15.83)	66(19.94)	
Worker	76(27.34)	80(24.17)	
Server	15(5.40)	34(10.27)	
Businessman	25(8.99)	28(8.46)	
Housework	35(12.59)	356(10.57)	
Teacher/government worker	28(10.07)	11(3.32)	
Others	55(19.78)	77(23.26)	
Family history with CHDs^a^			0.118
No	272(97.84)	321(95.54)	
Yes	6(2.16)	15(4.46)	
History of abortion^a^			0.025
No	185(66.55)	194(57.74)	
Yes	93(33.45)	142(42.26)	
History of adverse pregnancy^a^			0.67
No	127(45.68)	156(47.42)	
Yes	151(54.32)	173(52.58)	
Early pregnancy health^a^			< 0.001
Healthy	242(88.32)	197(60.80)	
Illness	32(11.68)	127(39.20)	
Fetus during pregnancy^a^			0.136
Normal	266(97.08)	317(94.63)	
Abnormal	8(2.92)	18(5.37)	
Folate supplements or multiple vitamin^a^			0.277
No	130(42.48)	158(46.75)	
Yes	176(57.52)	180(53.25)	
Smoking^a^			0.022
No	173(83.17)	302(89.88)	
Yes	35(16.83)	34(14.58)	
Drinking^a^			0.167
No	254(92.70)	297(89.46)	
Yes	20(7.30)	35(10.54)	
Pesticide exposure^a^			0.023
No	269(98.53)	319(95.22)	
Yes	4(1.47)	16(4.78)	
Drug exposure^a^			< 0.001
No	244(90.37)	247(75.77)	
Yes	26(9.63)	79(24.23)	

^a^Counting variables were described by frequency (percentage), and tested by *χ*^2^ test for differences between groups

^b^Quantitative data were described by mean ± SD, and tested by t-test for differences between groups

The genotype percentage values for MTHFR rs1801133 and rs1801131 have been given in Table 2 for the mothers. All the distributions were found to be in agreement with Hardy–Weinberg equilibrium. Logistic regression analyses showed that MTHFR rs1801133 was significantly increased their offspring CHDs risk compared with control group of mothers (OR 6.24, 95% CI 2.87–13.56 in Recessive model) (Table 2).

**Table 2:** Association of selected SNPs with offspring CHDs in mothers

Genotype	Control *N*(%)	Case *N*(%)	OR (95% CI)^a^	*P* value^a^
rs1801133				
CC	94(30.72)	108(31.95)	1	
CT	159(51.96)	161(47.63)	0.74(0.46–1.19)	0.211
TT	53(17.32)	69(20.41)	5.22(2.29–11.91)	< 0.001
Dominant			1.06(0.67–1.66)	0.812
Recessive			6.24(2.87–13.56)	< 0.001
Additive			1.59(1.13–2.22)	0.007
rs1801131				
AA	207(67.65)	228(67.46)	1	
AC	92(30.07)	91(26.92)	1.00(0.62–1.62)	0.996
CC	7(2.29)	19(5.62)	2.18(0.74–6.40)	0.157
Dominant			1.12(0.71–1.78)	0.624
Recessive			2.17(0.77–6.32)	0.155
Additive			1.19(0.82–1.74)	0.36

^a^Adjusted by gender of children, child-bearing age group, education, occupation, history of abortion, early pregnancy health, smoking, pesticide exposure an drug exposure

Table 3 shows the association of MTHFR rs1801133 and rs1801131 with plasma HCY level in mothers with offspring CHDs (12 unqualified samples of venous blood were excluded). The polymorphism of maternal MTHFR rs1801133 significantly increased plasma HCY level, especially with the homozygous mutation.

**Table 3:** Association of selected SNPs with plasma HCY level in mothers with offspring CHDs

Variables	*N*	Median	95%CI	*P*
rs1801133				< 0.001
CC	103	8.4	5.88–10.92	
CT	158	9.35	5.61–13.09	
TT	65	11.5	5.88–17.12	
rs1801133				< 0.001
CC	103	8.4	5.88–10.92	
CT/TT	223	9.8	5.26–14.34	
rs1801133				< 0.001
CC/CT	261	9	5.65–12.33	
TT	65	11.5	5.88–17.12	
rs1801131				0.652
AA	221	9.3	4.98–13.62	
AC	88	9.8	6.14–13.46	
CC	17	9.2	6.41–11.98	
rs1801131				0.691
AA	221	9.3	4.98–13.62	
AC/CC	105	9.5	5.97–10.03	
rs1801131				0.516
AA/AC	309	9.5	5.36–13.64	
CC	17	9.2	6.41–11.99	

## Discussion

The critical period of embryonic heart development is at 3–8 weeks of gestation. Environmental factors, as important as genetic factors, are causes of CHDs which mainly include the living environment, infection factors and drug effects of pregnant women [6–9]. In this study we found that gender of children, mothers’ child-bearing age, education, occupation, history of abortion, early pregnancy health, smoking, pesticide exposure and drug exposure were significantly associated with the risk of offspring CHDs susceptibility. The findings are consistent with those we observed in previous study.

MTHFR gene, catalyzes the conversion of 5,10-methylenetetrahydrofolate into 5-methyltetrahydrofolate. The latter is involved in the transformation of HCY to methionine, releasing tetrahydrofolate, which is decreasing the level of plasma HCY [10]. Therefore, the correlation between polymorphism of MTHFR gene and CHDs has become research focuses [11–13]. However, there is a great controversy reports about the relationship between the maternal MTHFR rsl801133 and rsl801131 polymorphism and their offspring CHDs [12, 14–16]. Some of them considered that rs1801133 and rs1801131 were related with the risk of CHDs [12, 13]. But some studies suggested that there is no association between the parents’ polymorphism of MTHFR rs1801133 and children’ CHDs [14]. Even there was a report identified MTHFR rs1801131 as a protective locus against CHDs [17]. In our previous study, we found the mothers who got a mutation of MTHFR rs1801131 CC genotypes had a 267% increase in risk of given birth of a CHDs children (OR 3.67, 95%CI 1.12–12.05). But in this study we found that the rs1801133 variant genotypes significantly increased their offspring CHDs risk by 524% (OR 6.24, 95% CI 2.87–13.56 in Recessive model) while no statistic difference between MTHFR rs1801131 polymorphism and offspring CHDs. The inconsistency of our results may be due to different adjustment variables, in the current study we additionally adjusted child-bearing age, education and smoking without adjusting family history with CHD, history of adverse pregnancy and fetus during pregnancy. Therefore, a larger sample size studies are required to achieve more robust results.

The plasma HCY level is another tricky problem. It was affected both by genetic and nutritional elements. In our previous study, we found that it was associated with offspring CHDs, and the critical value was 8.9 μmol/L determined by the ROC curve [18]. It is proved by nutrition studies that folic acid was key methyl donor in the plasma HCY metabolic pathway and VitB12 participates in the transfer of it. HCY could not be methylated to methionine by deficiency of folic acid or VitB12, which leads to hyperhomocysteinemia. However, the relationship between MTHFR rs1801133 and plasma HCY level were not very clear. It is reported that the polymorphism of MTHFR rs1801133 does not influence the level of plasma HCY. The mutation of MTHFR rs1801133 CT and TT genotypes is not found to be related to hyperhomocysteinemia [19] unless there is low level of folate occurred. In this study, we found that the polymorphism of maternal MTHFR rs1801133 significantly increased plasma HCY level, especially with the homozygous mutation, under the situation which there is no significant difference between the groups taking or not taking additional folate and multiple vitamins. We doubt that our crowd might not be lack of folate in Jiangsu Province, the south of China [20]. Thus, we come to the conclusion that the maternal MTHFR rs1801133 polymorphism might be a risk factor of offspring CHDs due to the hyperhomocysteinemia by abnormal metabolism of HCY.

In summary, besides the environmental factors, the maternal MTHFR rs1801133 and rs1801131 polymorphisms might be risk factors of offspring CHDs in different pathway which require further investigation in a large quantity of populations.

## Electronic supplementary material

Below is the link to the electronic supplementary material.

Supplementary Table S (DOC 31 kb)
