# Supplementary material for: Study on Maternal SNPs of MTHFR Gene and HCY Level Related to Congenital Heart Diseases
Source: Pediatr Cardiol. 2020 Nov 21;42(1):42–6. doi: 10.1007/s00246-020-02449-1 (PMC7864808; doi:10.1007/s00246-020-02449-1)
Supplement: Supplementary file 1 — Supplementary Table S (DOC 31 kb) [file 246_2020_2449_MOESM1_ESM.doc]

**Table S. Information for Primers and Probes by TaqMan Allelic Discrimination**

| Polymorphism | Sequence (5’-3’) |
| --- | --- |
| rs1801133 |  |
| Primer | F :CACAAAGCGGAAGAATGTGTCA |
|  | R : GACCTGAAGCACTTGAAGGAGAA |
| Probe | FAM-AAATCGGCTCCCGCA -MGB |
|  | HEX-TGAAATCGACTCCCG-MGB |
| rs1801131 |  |
| Primer | F :GGAGGAGCTGCTGAAGATGTG |
|  | R : TCTCCCGAGAGGTAAAGAACAAA |
| Probe | FAM-AAGACACTTTCTTCACTG -MGB |
|  | HEX-AGACACTTGCTTCAC-MGB |
